# Maternal early primary CMV infection with negative or equivocal CMV IgM

**DOI:** 10.1007/s10096-026-05525-1

**Published:** 2026-04-28

**Authors:** Vincent Portet Sulla, Rana Rafek, Elise Bouthry, Aude Simas, Abir Jadaoui, Lina Mouna, Claire Perillaud-Dubois, Christelle Vauloup-Fellous

**Affiliations:** 1 Division of Virology, National Reference Laboratory for viral perinatal infections, WHO Rubella National Reference Laboratory, Department of Biology and Genetics, Paris Saclay University Hospital, APHP, Paris, France; 2 Université Paris-Saclay, Inserm, CEA, “Immunological diseases, microbiology and innovative therapies” (IDMIT/UMR1184), Fontenay-aux-Roses & Le Kremlin-Bicêtre, France; 3 GRIG : Groupe de Recherche sur les Infections pendant la Grossesse, Paris, France; 4 Department of Virology, Angers University Hospital, 4 rue Larrey, Angers, 49933 France; 5 Virology Department, Sorbonne University, Saint-Antoine Hospital, AP-HP, Pierre Louis Epidemiology and Public Health Institute (iPLESP), INSERM 1136, Paris, France; 6 Laboratoire de virologie du Groupe Hospitalier Paris-Saclay, Hôpital Paul Brousse, 12-14 avenue Paul Vaillant Couturier, Villejuif, 94800 France

**Keywords:** CMV, Serology, IgM, Avidity, Pregnancy

## Abstract

**Background:**

Accurate diagnosis of primary cytomegalovirus infection (CMV PI) during pregnancy is critical for prognosis and clinical management, yet CMV-IgM may be transient or absent in the early phase of infection, leading to underdiagnosis. We aimed to determine the frequency and characteristics of negative or equivocal CMV-IgM results observed in confirmed recent CMV PI and to assess whether this diagnostic pitfall could be reduced.

**Methods:**

We retrospectively analysed 1,090 cases of CMV PI occurring within 2 months of infection onset in pregnant women referred to the French National Reference Laboratory for Viral Perinatal Infections between 2013 and 2025. CMV-IgG, CMV-IgM, and CMV-IgG avidity were assessed using the LIAISON^®^ XL platform, with additional avidity testing on VIDAS^®^. Recent infection was defined by IgG seroconversion and/or low IgG avidity (< 30% on VIDAS). We analysed the frequency, timing, and characteristics of negative or equivocal CMV-IgM results and evaluated the potential impact of pragmatic interpretation criteria.

**Results:**

Among 1,090 cases of early CMV PI, 2.6% (28/1,090) had negative or equivocal CMV-IgM results, including 1.7% (18/1,090) negative and 0.9% (10/1,090) equivocal results. CMV-IgM negativity was observed both within the first month [1.4% (9/664)] and between 1 and 2 months [2.1% (9/426)] after the onset of infection. Among IgM-negative patients, 39% (7/18) had low CMV-IgG levels on LIAISON^®^ XL (< 28 U/mL), consistent with very early seroconversion. In contrast, 61% (11/18) had CMV-IgG levels above twice the positivity threshold (> 28 U/mL), mimicking past immunity and representing a true diagnostic blind spot, accounting for 1.0% (11/1,090) of all infections. Considering CMV-IgM values between 15 and 18 U/mL as low-reactive results requiring further assessment, together with repeat serology when CMV-IgG levels were below twice the positivity threshold, would have reclassified 14 of 18 IgM-negative cases and reduced missed diagnoses from 1.7% (18/1,090) to 0.4% (4/1,090).

**Conclusion:**

Two complementary measures may improve detection of CMV-IgM-negative early infections while maintaining a manageable laboratory workload: repeat serology when CMV-IgG levels are very low, and further assessment of low-reactive CMV-IgM values between 15 and 18 U/mL. Nevertheless, a small residual diagnostic gap remains, highlighting the inherent limitations of CMV-IgM-based screening for recent CMV infection during pregnancy.

## Introduction

Cytomegalovirus (CMV) is the most common congenital infection worldwide, with an estimated global prevalence of 0.5–2% of live births [1, 2]. It represents a leading cause of non-genetic sensorineural hearing loss and neurodevelopmental impairment. One of the main risk factors for congenital infection is maternal primary CMV infection (CMV PI) during pregnancy. However, sequelae occur predominantly when infection is acquired in the peri-conceptional period or the first trimester of pregnancy [3–5]. In 2020, a randomized controlled trial demonstrated that oral valaciclovir (8 g/day) significantly reduces vertical transmission when administered after peri-conceptional or first-trimester infection [6]. These findings were further supported by an individual patient data meta-analysis showing a marked reduction in vertical transmission, together with lower rates of neonatal infection and pregnancy termination for severe fetal findings [7]. Therefore, accurate diagnosis and timing of CMV PI during pregnancy are critical, as infections occurring during the peri-conceptional period or first trimester carry a significant risk of transmission and long-term complications for the fetus, and as early diagnosis offers a therapeutic window to reduce mother to fetus transmission [3, 5, 7]. As recommended by the European Congenital Cytomegalovirus Initiative (ECCI), serological assessment during pregnancy relies on CMV-IgG and CMV-IgM testing, with CMV-IgG avidity testing used to help date infection when both antibodies are detected [8]. However, CMV-IgM is an imperfect marker of recent primary infection. Cross-reactivity and prolonged persistence may limit its specificity for dating infection, and important inter-assay differences have been reported. In some cases, CMV-IgM may already be undetectable during the early phase of primary infection, depending on the assay used [9–11]. This variability creates a diagnostic blind spot in the first weeks of infection, when IgM may be negative or equivocal despite recent CMV PI. In our experience, this includes cases with documented IgG seroconversion without detectable IgM, as well as cases with low-avidity IgG but persistently negative IgM results. Understanding and preventing this diagnostic pitfall is essential to improve CMV screening accuracy in pregnancy. Therefore, this study aimed to characterize the frequency of negative or equivocal CMV-IgM results in early CMV PI (< 2 months), and to suggest practical laboratory strategies to avoid misclassification.

## Materials and methods

### Study population and design

We conducted a retrospective data analysis of CMV serological results from samples collected from pregnant women and referred to the French National Reference Laboratory for Perinatal Viral Infections (Division of Virology, Department of Biology and Genetics, Paris-Saclay University Hospital, AP-HP, France) between 2013 and July 2025.

### Ethics

As part of routine care in our hospital, patients are informed that, unless they formally object, their medical data may be used for research purposes. No patient objected. All data were analyzed anonymously. Given the retrospective design and the use of anonymized routine-care data, formal written informed consent was not required. The study was conducted in accordance with the Declaration of Helsinki and with the applicable French regulations.

### Serological testing

All tests were performed at the time of initial testing in our laboratory, and no re-testing of stored specimens was performed for this study. All assays were performed using the LIAISON XL platform (DiaSorin^®^, Saluggia, Italy) for CMV-IgG, CMV-IgM, and CMV-IgG avidity. In our reference laboratory setting, CMV-IgG avidity testing is not restricted to samples with positive or equivocal CMV-IgM on LIAISON XL. Because serum samples are referred from many laboratories across France for expert CMV serology interpretation, often after initial testing on other analytical platforms, some samples may show positive or equivocal CMV-IgM on another assay despite negative CMV-IgM on LIAISON XL. For this reason, CMV-IgG avidity is performed whenever recent primary infection is clinically or biologically suspected, including in samples with negative CMV-IgM on LIAISON XL. When the LIAISON IgG avidity index was ≥ 0.4, the case was classified as CMV PI > 3 months. When the LIAISON IgG avidity index was < 0.4, an additional IgG avidity test was performed using the VIDAS platform (bioMérieux^®^, Craponne, France) to further refine infection timing [12]. This reference-laboratory workflow explains how recent CMV primary infections with negative CMV-IgM on LIAISON XL could be identified even in the absence of documented seroconversion. According to the manufacturer’s cut-offs for LIAISON XL (DiaSorin), CMV-IgM negativity is defined as < 18 U/mL, and equivocal results as values between 18 and 22 U/mL. “Uncertain immune status” referred to cases with low LIAISON XL CMV-IgG concentrations, defined here as below twice the positivity threshold (14 U/mL), i.e. <28 U/mL (< 2× cut-off) (Table 1) according to the national consensus algorithm established by French Herpesviruses National Reference Center and National Reference Laboratory for Viral perinatal infections.

### Definition of recent CMV primaryinfection

We deliberately restricted the analysis to primary-infections occurring less than two months prior to sample collection, in order to focus on the earliest phase of CMV PI when CMV-IgM are still expected to be positive. In our study, CMV PI < 2 months was defined by at least one of the following criteria: CMV-IgG avidity < 30% on the VIDAS assay with CMV-IgG ≥ 12 AU/mL on VIDAS (i.e., at least twice the VIDAS IgG positivity threshold of 6 AU/mL), and/or documented CMV-IgG seroconversion. To classify infections as occurring within < 1 month, we used very low avidity values (< 20% on VIDAS) or evidence of recent IgG seroconversion. Cases with CMV-IgG levels < 12 AU/mL on the VIDAS assay (i.e., less than twice the VIDAS positivity threshold) were excluded to avoid misclassification due to uncertain immune status affecting avidity interpretation (Table 1).

**Table 1:** Criteria used to define early CMV PI and uncertain immune status

Classification	Definition	VIDAS CMV-IgG avidity	Additional notes
CMV PI < 1 month	Recent seroconversion and/or very low avidity	< 20%	Only cases with CMV-IgG ≥ 12 AU/mL on VIDAS (≥ 2× positivity threshold of 6 AU/mL) were retained
CMV PI 1–2 months	Recent seroconversion and/or low avidity	20–30%	Only cases with CMV-IgG ≥ 12 AU/mL on VIDAS (≥ 2× positivity threshold of 6 AU/mL) were retained
Uncertain immune status	Low CMV-IgG concentration on LIAISON XL or VIDAS	—	- LXL CMV-IgG < 28 U/mL (< 2× LIAISON XL CMV-IgG positivity threshold of 14 U/mL) - VIDAS CMV-IgG < 12 AU/mL (< 2× VIDAS CMV-IgG positivity threshold of 6 AU/mL)

### Data analysis

We identified all patients with confirmed CMV PI < 2 months and classified them according to CMV-IgM results. Cases were stratified according to the estimated time since primary-infection (< 1 month vs. 1–2 months). Descriptive analyses were performed to determine the frequency of negative and equivocal IgM results. In addition, we evaluated the potential impact of considering CMV-IgM values between 15 and 18 U/mL as low-reactive rather than strictly negative on patient classification and on the additional IgG avidity testing workload associated with this approach. Finally, in cases of negative CMV-IgM, CMV-IgG levels were reviewed to assess antibody dynamics and exclude potential misclassification. This approach was designed both to quantify the occurrence of negative or equivocal CMV-IgM results in early CMV PI and to identify the testing conditions under which these diagnostic pitfalls are most likely to occur.

## Results

### Frequency of negative or equivocal CMV-IgM results

**Fig. 1:**
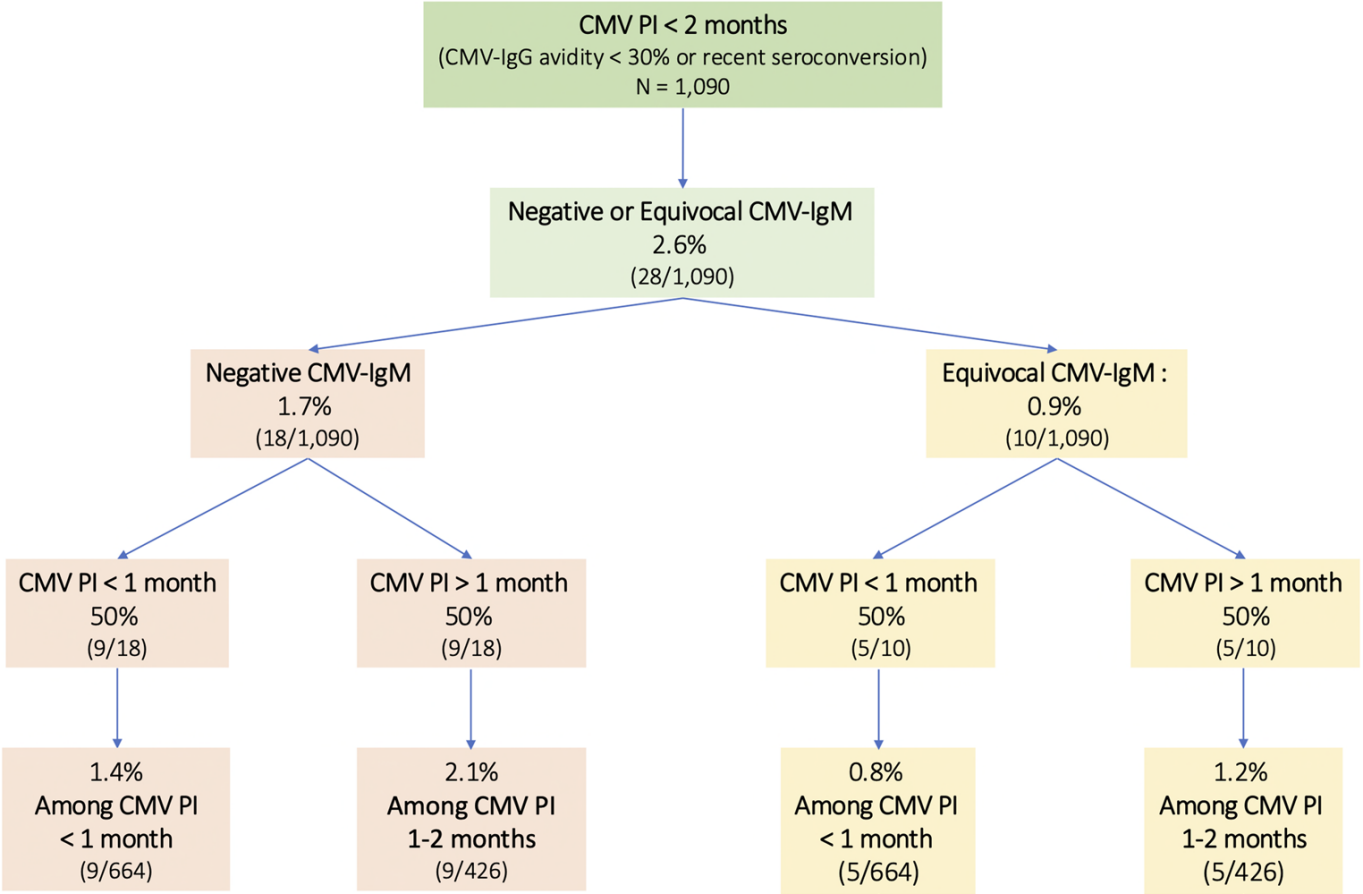
Rate of negative or equivocal CMV-IgM in early CMV PI (< 1 month and 1–2 months)

Between 2013 and mid-2025, we identified 1,090 cases of CMV PI including 664 < 1 month and 426 between 1 and 2 months after onset of infection (Fig. 1). Overall, 2.6% (28/1,090) exhibited negative or equivocal CMV-IgM results on LIAISON XL, including 1.7% (18/1,090) negative and 0.9% (10/1,090) equivocal (Fig. 1). These 28 cases corresponded to a total of 74 serum samples (follow-up specimens).

The distribution was similar across both time frames, with 9 negative and 5 equivocal cases observed within the first month and the same numbers between one and two months. These corresponded to proportions of 1.4% (9/664) negative CMV-IgM and 0.8% (5/664) equivocal CMV-IgM within the first month, and 2.1% (9/426) negative CMV-IgM and 1.2% (5/426) equivocal CMV-IgM at 1–2 months (Table 2; Fig. 1).

**Table 2:** Timing distribution of early CMV PI with negative or equivocal IgM results

	Total CMV PI < 2 months	CMV PI < 1 month	CMV PI 1–2 months
Number of cases	1090	664	426
Negative CMV-IgM	1.7% (18/1090)	1.4% (9/664)	2.1% (9/426)
Equivocal CMV-IgM	0.9% (10/1090)	0.8% (5/664)	1.2% (5/426)

### Performance of CMV-IgM detection

Based on our cohort of 1,090 confirmed CMV PI < 2 months, the apparent sensitivity of CMV-IgM detection on the LIAISON XL platform was 98.3% (1,072/1,090) overall, when equivocal results were considered reactive. When stratified by infection timing, apparent sensitivity reached 98.6% (655/664) during the first month and 97.9% (417/426) during the second month post-infection. Specificity could not be assessed in our dataset, as only confirmed CMV PI were included.

### Role of CMV-IgG levels in identifying recent infection

**Fig. 2:**
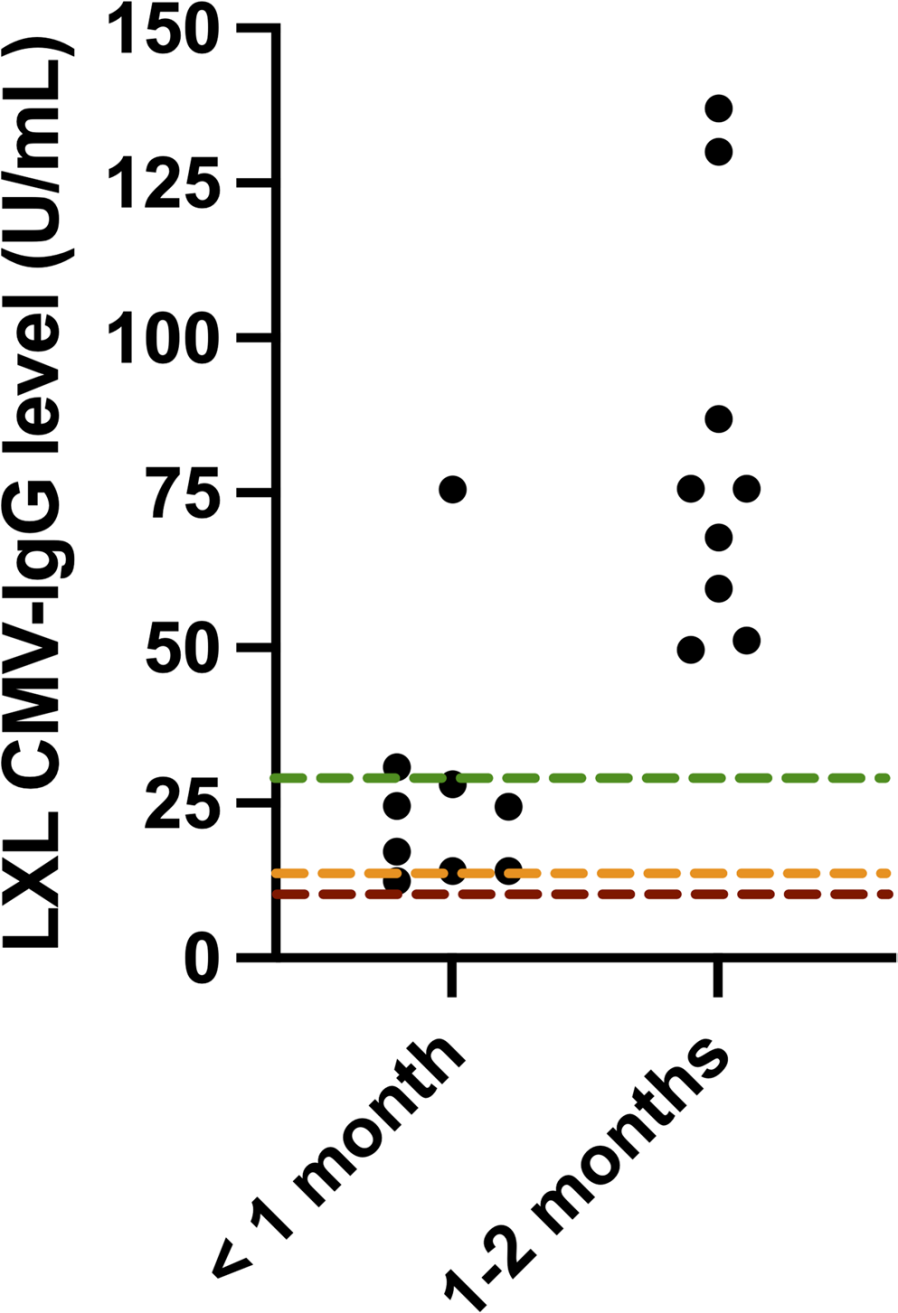
Distribution of CMV-IgG levels in early CMV PI and identification of low IgG profiles

The CMV-IgG level itself may serve as a useful indicator for patient classification. In our cohort, seven patients displayed a CMV IgG-positive/IgM-negative profile with low IgG concentrations (< 28 U/mL on the LIAISON XL), representing 39% (7/18) of IgM-negative cases within 2 months of infection. These profiles likely reflect very early seroconversion, where IgG becomes detectable before IgM reaches measurable levels by the assay. As shown in Fig. 2, most patients with low IgG levels (< 2 × positivity threshold) and negative IgM were classified within the < 1-month infection group, whereas patients with IgG >28 U/mL were predominantly classified within the 1–2-month group. Among these seven patients, four later developed detectable IgM (15–60 days after the initial sample) and six showed rising IgG levels, confirming ongoing infection. One patient did not have a follow-up sample, but her CMV-IgM level was 16.9 U/mL, slightly below the manufacturer’s cut-off of 18 U/mL. Conversely, eleven IgM-negative patients had IgG levels > 28 U/mL (2 within < 1 month and 9 within 1–2 months post-infection), mimicking “past immunity”. Without IgG avidity testing, these cases would likely be overlooked, representing a small but significant diagnostic blind spot [1.0% (11/1,090) of all infections].

Distribution of CMV-IgG levels (U/mL) measured on the LIAISON XL platform in patients with early CMV PI (< 1 month and 1–2 months). The red dashed line indicates the manufacturer’s equivocal threshold (12 U/mL), the orange dashed line marks the positivity threshold (14 U/mL), and the green dashed line represents twice the positivity threshold (28 U/mL), used to define low CMV-IgG levels (< 2 × cut-off) suggestive of uncertain immune status.

### Distribution of CMV-IgM values and impact of threshold adjustment

**Fig. 3:**
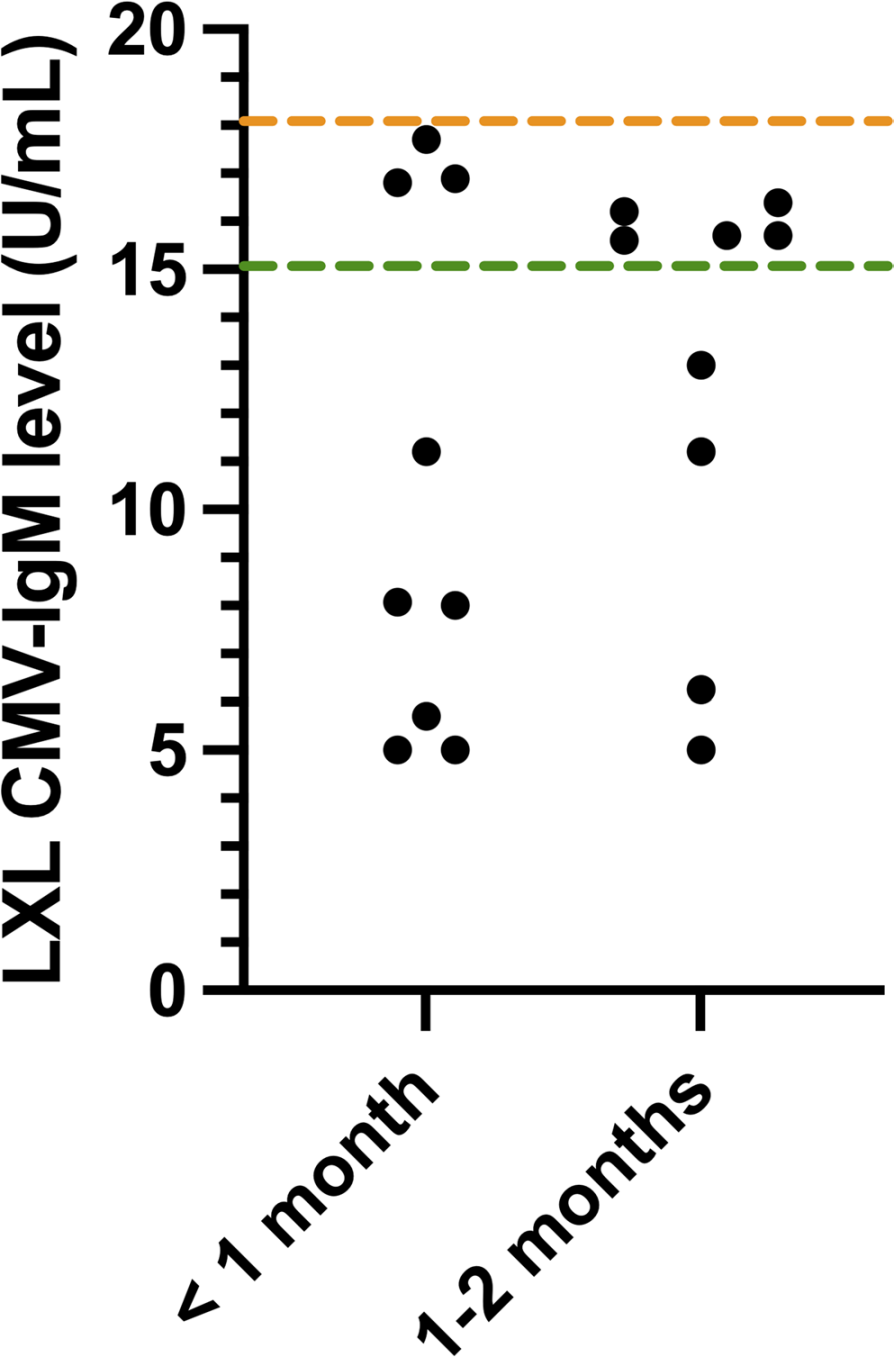
Distribution of negative CMV-IgM levels in early PI and impact of threshold adjustment

According to the manufacturer’s cut-offs, CMV-IgM negativity is defined as < 18 U/mL while equivocal results fall between 18 and 22 U/mL. Among IgM-negative samples, three were below the assay’s detection limit (< 5 U/mL), while the remaining values ranged from 5.0 to 17.7 U/mL, with a median of 12.1 U/mL (IQR 6.7–16.1). Equivocal values displayed a distribution with a median of 20.0 U/mL (IQR 18.8–20.5) and values ranging from 18.0 to 21.8 U/mL. If CMV-IgM values between 15 and 18 U/mL were considered low-reactive rather than strictly negative, eight patients (44.4%; 8/18) initially classified as negative would have been reclassified (Fig. 3). In practice, this adjustment would reduce the number of samples classified as strictly negative (from 18 to 10) and proportionally increase the number of CMV-IgG avidity tests performed, since each equivocal CMV-IgM result triggers an avidity assay. For example, in 2024, our laboratory analysed 1,670 serum samples collected from pregnant women and performed 505 CMV-IgG avidity tests due to positive or equivocal CMV-IgM results. Such an approach would have resulted in 91 additional avidity tests (total 596), corresponding to an 18% increase in testing workload, or roughly 1.7 additional tests per week.

Distribution of CMV-IgM values (U/mL) measured on the LIAISON XL platform in patients with early CMV PI (< 1 month and 1–2 months). The orange dashed line indicates the manufacturer’s negativity threshold (18 U/mL). The green dashed line marks the proposed adjusted threshold (15 U/mL) allowing reclassification of low-reactive samples as equivocal.

## Discussion

In this study, we assessed the frequency and characteristics of negative or equivocal LIAISON^®^ XL CMV-IgM results during the early phase of CMV PI in pregnancy. By restricting inclusion to infections occurring within two months of onset, as defined by a stringent low-avidity criterion on VIDAS (< 30%) and/or documented IgG seroconversion, we focused specifically on the time window in which CMV-IgM is generally expected to be detectable. Among 1,090 confirmed CMV PI < 2 months, 1.7% (18/1,090) presented with negative CMV-IgM and 0.9% (10/1,090) with equivocal results on the LIAISON XL assay. When equivocal results are interpreted as reactive, this corresponds to an overall apparent detection rate of 98.3% (1,072/1,090), including 98.6% (655/664) in the first month and 97.9% (417/426) in the second. Although this proportion is high, it still leaves a small but clinically relevant diagnostic blind spot in a high-risk context such as pregnancy.

### Limits of CMV-IgM detection in early CMV primary infection

Currently available CMV-IgM assays generally show good analytical performance, but CMV-IgM kinetics vary substantially over time and between individuals, which may complicate the serological dating of primary infection [13]. Early undetectable CMV-IgM has nevertheless been documented. Revello et al. showed that a small proportion of samples collected within 30 days after infection were already CMV-IgM negative, namely 2.4% with the Elecsys^®^ assay and 3.6% with the ETI-CYTOK-M reverse PLUS ELISA [9]. Sarasini et al. analysed 426 CMV PI in pregnant women and found that 4.7% already had undetectable CMV-IgM within three months of infection on the LIAISON^®^ CMV IgM II assay [11]. Notably, in their cohort, all LXL IgM-negative cases occurred after 30 days post-infection, whereas our data, with the same assay, show that CMV-IgM negativity can already occur within the first 30 days, extending this phenomenon to an even earlier diagnostic window. More broadly, Daiminger et al. compared eight commercial CMV-IgM assays in 100 serum samples from 39 pregnant women and highlighted marked inter-assay variability in both early detection and later CMV-IgM persistence [14]. In their series, some assays showed incomplete CMV-IgM detection during the first 12 weeks after infection, whereas several assays, including LIAISON^®^, remained positive in more than 85% of samples beyond 20 weeks. Such prolonged persistence substantially limits the specificity of CMV-IgM as a marker of recent infection and reduces its practical value for identifying true CMV primary infection in pregnancy. Together, these comparative data indicate that no single CMV-IgM assay simultaneously offers optimal early sensitivity, specificity, and appropriately limited IgM persistence.

### The residual diagnostic blind spot of CMV-IgM–based screening

Our findings complement these previous studies by documenting, in a large real-life cohort, a small subset of early CMV PI in which IgM testing fails to suggest CMV PI. An important contribution of our study is the characterization of IgG-positive/IgM-negative profiles with low IgG levels. In our cohort, seven IgM-negative patients had IgG concentrations below twice the positivity threshold (< 28 U/mL on LIAISON XL), combined with very low VIDAS avidity and/or documented seroconversion. These cases represented 39% (7/18) of all IgM-negative infections within 2 months. Among them, four later developed detectable CMV-IgM (within 15–60 days after the initial sample), and six showed a significant rise in IgG levels, confirming ongoing seroconversion. In such cases, where low IgG levels and negative IgM are followed by a significant rise in IgG levels on a subsequent sample, a recent CMV PI should be suspected. By contrast, in 61% (11/18) of IgM-negative CMV PI, CMV-IgG levels were already above twice the positivity threshold (> 28 U/mL), mimicking the serological profile of past immunity. In such cases, no routine parameter suggests a recent infection and IgG avidity testing would not typically be requested. These profiles therefore represent a genuine diagnostic blind spot, accounting for approximately 1% (11/1,090) of all early CMV PI and illustrate the true pitfall of CMV-IgM–based screening strategies in pregnancy.

### Clinical and laboratory implications

As demonstrated by Fourgeaud et al., triggering CMV-IgG avidity testing only when CMV-IgM is detectable remains an efficient screening strategy during pregnancy [15]. Their study showed that this targeted approach achieved 91.6% sensitivity for diagnosing CMV primary infection, compared with 83% for systematic avidity testing of all CMV-IgG-positive samples. Performing avidity testing on every CMV-IgG-positive sample would be unrealistic in most laboratories because of the associated costs and workload. In this context, our findings support pragmatic complementary criteria to reduce the risk of missing very recent infections. First, CMV-IgG levels below twice the positivity threshold (< 2× cut-off) in CMV-IgM-negative samples should not be interpreted as evidence of past infection or reassuring immunity. Rather, these profiles should be considered as an uncertain serological status prompting repeat serology after 1–2 weeks. If follow-up testing shows rising CMV-IgG levels, recent CMV primary infection should be suspected and additional investigations should be performed according to the clinical context, including CMV-IgG avidity testing and/or virological investigations. Second, considering LIAISON^®^ XL CMV-IgM values between 15 and 18 U/mL as low-reactive results requiring further assessment would prompt additional CMV-IgG avidity testing. In our study, 44.4% (8/18) of primary infections with initially negative CMV-IgM had values ≥ 15 U/mL. Based on our routine activity in 2024, such an approach would have resulted in 91 additional avidity tests over one year, corresponding to an 18% increase (91/505) and approximately 1.7 additional tests per week. In our reference laboratory setting, this additional workload appears manageable. Importantly, this proposal should be understood as a pragmatic interpretation strategy in a high-risk setting rather than as a formal modification of the manufacturer’s validated assay cut-off. Altogether, these pragmatic adjustments may improve early CMV infection detection while maintaining the overall efficiency of current CMV-IgM-based screening algorithms. Applied together, repeat serology for low CMV-IgG profiles and pragmatic interpretation of CMV-IgM values between 15 and 18 U/mL as requiring further assessment would have reclassified 14 of the 18 initially CMV-IgM-negative cases as recent infections (7 with low CMV-IgG levels and 8 with CMV-IgM ≥ 15 U/mL, with one patient fulfilling both criteria), thereby reducing the apparent rate of missed cases from 1.7% (18/1,090) to 0.4% (4/1,090). Finally, equivocal CMV-IgM values, although rare [0.8% (5/664) in the first month and 1.2% (5/426) in the second], may also be associated with recent CMV primary infection. They should therefore systematically prompt CMV-IgG avidity testing to clarify infection timing.

### Study limitations

This study has several limitations. First, it was retrospective and based on samples referred to a national reference laboratory, which may have enriched the cohort for diagnostically complex or atypical cases and may limit generalizability to unselected first-line screening settings. Second, infection dating relied on serological criteria, mainly avidity testing and seroconversion, which remain indirect estimates of infection timing. Although we used a stringent VIDAS avidity threshold to define recent infection, no single assay perfectly correlates with the true timing of infection. To minimize potential misclassification, we excluded cases with CMV-IgG levels < 12 AU/mL on the VIDAS assay, as avidity results are unreliable when IgG concentrations are very low; this conservative approach may, however, have slightly underestimated the true frequency of very early CMV primary infection without detectable IgM. Third, our findings are based on a single CMV-IgM analytical platform (LIAISON^®^ XL) and may not be directly extrapolated to other assays. Finally, the pragmatic interpretation of CMV-IgM values between 15 and 18 U/mL discussed here should be understood as a laboratory-based strategy for high-risk settings rather than as a validated manufacturer-level cut-off modification.

## Conclusions

Altogether, our results confirm that negative or equivocal CMV-IgM results occur in 2.6% (28/1,090) of confirmed early CMV primary infections, including 1.4% (9/664) with negative CMV-IgM within the first month and 2.1% (9/426) within the second month after infection. This subset represents a genuine diagnostic pitfall in routine prenatal screening. To minimize misclassification, equivocal IgM results should prompt avidity testing, IgM-negative samples with low IgG levels should be retested after a short interval, and low-reactive CMV-IgM values between 15 and 18 U/mL may warrant further assessment in pregnancy screening settings. Implementing these measures may reduce misclassification, although a small residual diagnostic blind spot of approximately 0.4% (4/1,090) appears to remain.

## Data Availability

The data are available upon request.
